# Polyinosinic:polycytidylic acid causes epithelial-mesenchymal transition via BAFF expression in Beas-2B human bronchial epithelial cells

**DOI:** 10.7150/ijms.129414

**Published:** 2026-05-11

**Authors:** Yatham Naga Satya Mani, Dae Heum Yun, Eun-Yi Moon

**Affiliations:** Department of Integrative Bioscience and Biotechnology, Sejong University, Seoul 05006, Rep. of Korea.

**Keywords:** polyinosinic-polycytidylic acid (Poly (I:C)), B cell activating factor, reactive oxygen species, epithelial-mesenchymal transition

## Abstract

The bronchial epithelium acts not only as the primary physical barrier but also as an active sensor that responds to exogenous materials such as bacteria and viruses, by producing various cytokines including B-cell activating factor (BAFF). Although BAFF is a well-known protein in B-cell functions, its role in bronchial cell function remains undefined. Polyinosinic-polycytidylic acid (Poly (I:C)), a synthetic double-stranded RNA, serves as a model for viral infection that binds to toll-like receptor (TLR) 3 to trigger intracellular signal pathways. In this study, we investigated the effect of Poly (I:C)-induced BAFF expression on airway cell migration using Beas-2B human bronchial epithelial cells. Poly (I:C) increased BAFF expression and cell migration, along with the increased expression of N-cadherin, Vimentin and Slug (SNAI2), which are three primary markers for epithelial-mesenchymal transition (EMT). Cell migration was attenuated by small interfering RNA (siRNA) against BAFF, which also inhibited the expression of these EMT markers. Phosphorylation of c-Jun N-terminal kinase (JNK) was enhanced by Poly (I:C) and inhibited by SP600125, JNK inhibitor, leading to a decreased expression of BAFF and aforementioned EMT markers. Poly (I:C) increased reactive oxygen species (ROS), resulting in a ROS-dependent up-regulation of antioxidants (Catalase, superoxide dismutase (SOD)1 and heme oxygenase (HMOX)1) and Nrf2. Pre-treatment with N-acetylcysteine (NAC), ROS scavenger, inhibited Nrf2 activation and JNK phosphorylation. The increase in Nrf2 levels induced by Poly (I:C) was also attenuated by SP600125. Additionally, while NAC treatment inhibited BAFF expression, it caused little change in the expression of the three EMT markers. Increased BAFF expression was confirmed by Nrf2 binding to the BAFF promoter or an increase in the luciferase activity of BAFF promoter co-transfected with Nrf2 plasmids. Treatment with recombinant BAFF protein also increased cell migration and EMT marker expression. Taken together, our results demonstrate that Poly (I:C) promotes a regenerative migration of bronchial epithelial cells by inducing BAFF expression through the ROS-dependent JNK-Nrf2 signaling axis.

## Introduction

B cell-activating factor (BAFF) plays a significant role to regulate B cell development, differentiation, maturation and survival 1 and to maintain B cells in peripheral lymphoid organs 2. BAFF is produced by immune cells including macrophages, dendritic cells and neutrophils 3, 4 or by non-immune cells including synoviocytes 5, 6, human intestinal epithelial cells 7, salivary gland 8 and airway cells 9. BAFF binds to its receptors, BAFF-R, TACI and BCMA on B cells and supports their proliferation, maturation and antibody secretion 10. However, it may not be defined the role of BAFF function in other types of cells yet, including airway epithelial cells.

Airway epithelium serves as the first line of defense against airborne pathogens, acting not only as a physical barrier but also in modulating immune responses 11-13. Upon exposure to toxic agents including bacteria or viruses, the respiratory epithelium is frequently the site of injury and plays a role in airway repair and remodeling by changing the local environment 14-16. Migration of neighboring cells is an important component for the rapid repair of damaged airway epithelium 17, 18. However, little information has been reported about the role of BAFF in airway epithelium cell migration.

Polyinosinic:polycytidylic acid (Poly (I:C)), a synthetic double stranded RNA, is widely used to modulate viral infections 15, 16. Poly (I:C) induces innate immune responses through TLR3, a pattern recognition receptor (PRR), abundantly expressed in various epithelial cell models including airway epithelial cells 19, 20. Poly (I:C) activates airway epithelium cells to produce type I interferon (IFN), especially IFN-β, leading to BAFF expression 9, 21. Poly (I:C) induces apoptosis via the rapid secretion of chemokines, which may have detrimental effects on the damaged epithelium 22. In addition, Poly (I:C) stimulates retinoic acid synthesis and signaling, thus promoting the healing 23 of mammalian skin wounds 24 and damaged neonatal heart 25. Poly (I:C) increased the phosphorylation of various kinases including p38 MAPK, extracellular signal-regulated kinase (ERK), c-Jun N-terminal kinases (JNK) and protein kinase B (Akt) in microglial cells 26. However, it has not been known about whether BAFF plays a potential role to influence epithelial plasticity and how Poly (I:C) modulates BAFF expression. In addition, the signaling molecules that regulate airway cell migration in response to Poly (I:C) are thus far unknown, as is their mechanism of action.

It has been reported that Epithelial-mesenchymal transition (EMT) plays an important role in lung physiology functioning in the routine repair of epithelial defects to maintain the integrity of the respiratory tract lining 27. EMT is a dynamic and reversible biological process to change epithelial plasticity. Epithelial cells in EMT undergo a phenotypic transition by acquiring migratory and invasive characteristics of mesenchymal cells 28, 29. EMT play a crucial role in embryonic development, tissue repair and pathogenesis of fibrosis and cancer metastasis 30. In addition, EMT provides epithelial cells the ability to migrate into injury sites 31 and mesenchymal-like cells secrete extracellular matrix proteins such as collagen, fibronectin and laminin for new tissue formation 32. EMT also increases cellular plasticity and allows cells to replace damaged specialized cells in organs such as the skin, kidneys, liver and intestine 33. In addition, EMT contributes to pathogenesis of gentamicin-induced nephrotoxicity 34 or fibrosis 35, 36. EMT is characterized by down-regulation of epithelial markers like E-cadherin and up-regulation of mesenchymal markers like N-cadherin, Vimentin and fibronectin 37. EMT is initiated by external stimuli such as growth factors, pro-inflammatory cytokines, and viral mimics like Poly (I:C) 38. However, it is unknown about whether Poly (I:C) contributes to the regeneration of airway epithelial damage by EMT through BAFF expression.

Oxidative stress is resulted in the accumulation of reactive oxygen species (ROS) 39. ROS are chemically reactive molecules that play critical roles in cellular signaling and homeostasis 40. ROS activate multiple signaling pathways, including MAPK pathways like p38, JNK, and ERK 41. ROS also activate Nrf2 by disrupting the interaction between keap1 and Nrf2 41, 42. Free Nrf2 translocates into the nucleus and transactivates the antioxidant response elements (AREs) of many cytoprotective antioxidant genes 43. The switching on and off of Nrf2 protects cells against free radical damage, prevents apoptosis, and promotes cell survival. 44. Oxidative stress generated by external stimuli also promotes the expression of transcription factors such as Slug (SNAI2), Snail, Twist, and ZEB1 to regulate EMT markers 45-47 or to cause oxidative damage to DNA and genomic instability 48. However, little is known about whether Poly (I:C)-mediated ROS-Nrf2 axis contributes to BAFF expression leading to EMT in airway epithelial cells.

In our study, we investigated the association between airway epithelium cell migration induced by TLR3 activation and BAFF expression using Poly (I:C) and Beas-2B human bronchial epithelial cells. We evaluated the expression of EMT molecules to determine the potential role of BAFF in promoting EMT in BEAS-2B cells in response to viral-like stimuli. Our results demonstrate the ability of Poly (I:C)-mediated BAFF expression to induce the loss of epithelial cell integrity through the increase in ROS production and the activation of JNK/Nrf2 signaling.

## Materials and Methods

### Reagents

Polyinosinic:polycytidylic acid (Poly (I:C), P0913) and N-acetyl-L-cysteine (NAC, A9165) were purchased from Sigma-Aldrich Co. (St. Louis, MO, USA). 2',7'-dichlorodihydrofluorescein diacetate (DCF-DA, D399) was obtained from Thermo Fisher Scientific Inc. (Waltham, MA, USA). Anti-β-tubulin antibody (3G^) was from Abbkine Scientific Co., Ltd (Waltham, MA, USA). Anti-Nrf2 antibody (16396-1-AP) was from Proteintech Group Inc. (Rosemont, IL, USA). Antibodies to BAFF were purchased from Sigma Chemical Co. (PRS2221, St. Louis, MO, USA) or Merk Millipore (AB16530, Darmstadt, Germany). Small interference (si) RNA for BAFF was synthesized by Bioneer (Daejeon, Korea). Recombinant human BAFF protein was obtained from R&D System Inc. (Minneapolis, MN, USA). Polyethylenimine (PEI) was purchased from Polysciences, Inc. (Warrington, PA, USA). Lipofectamine^®^ 2000 was obtained from Thermo Fisher Scientific (Carlsbad, CA, USA). Except where indicated, all other materials are obtained from the Sigma Chemical Co. (St. Louis, MO, USA).

### Cell culture

Beas-2B human bronchial epithelial cells (ATCC # CRL-9609) were obtained from Korea research institute of bioscience and biotechnology (KRIBB) cell bank (Daejeon, Rep. of Korea). Cells were adapted and cultured as monolayers in Dullecco's modified Eagle's medium (DMEM) with supplement of 10% fetal bovine serum (FBS) (GIBCO, Grand Island, NY, USA), 2 mM L-glutamine, 100 units/ml penicillin/streptomycin (GIBCO, Grand Island, NY, USA). Cells were incubated at 37 ^o^C in a humidified atmosphere of 5% CO_2_ maintenance.

### Cell adaptation

Beas-2B cells were originally maintained in the coated dishes with FBS-free BEBE containing BEBM^TM^ bronchial epithelial cell growth basal medium along with all the additives, BEGM^TM^ kit (Lonza/Clonetics Co., Basel Switzerland). Beas-2B cells were adapted by replacing BEBE to DMEM supplemented with 10% FBS following a protocol. Briefly, culture medium was sequentially exchanged by feeding the fresh BEBE containing 25%, 50%, 75% and 100% DMEM supplemented with 10% FBS every 24 h. After 5 days, cells were sub-cultured into uncoated dishes with DMEM containing 10% FBS. The cells were fully adapted by sub-culture up to 40 times after the start of adaptation. All experiments were performed with Beas-2B cells at 40^th^-60^th^ passage modified from previous reports 49-51.

### Wound healing assay

Cell migration was measured as described previously, with minor modifications 6. Briefly, when MH7A cells reached confluence in a 35-mm culture dish (Corning, NY, USA), three wound lines were made by scratching the cellular layer with a plastic yellow pipette tip. Floating cells were washed out, and fresh medium was added. Cells were then incubated at 37 °C in a humidified atmosphere of 5% CO_2_ maintenance. Narrowing of the wound was then monitored using a phase-contrast microscope beginning 6 h after the scratch. The empty area of the wound at each time point was then quantified using NIH image analysis software (ImageJ, version 1.34n), and compared with that in the initiation of cell migration.

### Boyden chamber assay

Experiments were conducted using a 24-well Transwell system (6.5mm Transwell (#3422), Corning, NY, USA) with each well separated by a microporous 10 μm thin transparent polycarbonate membrane (8 μm pore size) into an upper (“insert”) and a lower chamber (“well”). A volume of 100 μL containing 15,000 cells were plated to each insert and 600 μL medium was added to the wells. Cells in 'insert' were allowed to migrate for 12 h. Then, cells were fixed and stained in a 20% methanol/0.1% crystal violet solution for 15 min at room temperature, followed by washing 'insert' with water to remove redundant staining. Cells on upper part of 'insert' membrane were wiped out. Cells migrated onto lower part of 'insert' membrane were photographed, counted and presented as bar graph 6.

### Measurement of BAFF gene transcriptional activity

The 1kb upstream segment of the BAFF promoter had been previously cloned into pGL3-basic vector 4. BEAS-2B cells were transfected with pGL3-BAFF-Luc plasmid and cell lysates were prepared to assess BAFF gene transcriptional activity by the incubation with luciferase substrate purchased from Promega Co. (Madison, WI, USA). Then, luminescence was measured by using Glomax® Discover Microplate Reader (Promega, Madison, WI, USA).

### Transfection with small interference (si) RNA or plasmid DNA

When cells reached 70-80% confluency in 6-well plate, the medium was replaced with serum-free medium. Cells were respectively transfected with small interference (si) RNA or plasmid DNA by using Lipofectamine^®^ 2000 or PEI transfection reagent following the manufacturer's protocol. Briefly, 20 pmole of each siRNA, 1 μg of plasmid DNA and 1 or 3 μl of Lipofectamine^®^ 2000 were mixed respectively after each was diluted in 50 uL serum-free medium. Mixture was allowed to stand for an additional 20 min and added to the cells. Then, cells treated with Lipofectamine^®^ 2000 mixture were incubated for 4 h and the medium was replaced with complete DMEM containing 10% FBS, penicillin/streptomycin, and L-glutamine. No medium change is required for the cells treated with PEI mixture. After incubated for 24 h, the cells were used for other experiments such as RT-PCR, western blot analysis and assay to measure cell migration and BAFF gene transcriptional activity.

### Measurement of reactive oxygen species

The reactive oxygen species (ROS) levels were assessed by incubating cells with or without 10 µM of 2',7'-dichlorofluorescin diacetate (DCF-DA) (Molecular Probe, Eugene, USA) at 37 °C for 20 minutes. Fluorescence intensity was measured using Glomax® Discover Microplate Reader (Promega, Madison, WI, USA).

### Reverse transcriptase polymerase chain reaction (RT-PCR)

Total RNA extraction was performed using NucleoZOL (MACHEREY-NAGEL GmbH & Co. KG, Duren, Germany). Using oligo-dT18 primers and reverse transcriptase, cDNA was synthesized from 1μg of total RNA (Bioneer, Daejeon, Korea) in a total volume of 21 µL. PCR amplification was performed using 1 µL of the first-strand cDNA as a template and 10 pmol of specific primers amplified with Taq DNA polymerase. PCR amplification was carried out using primers specific to BAFF (sense: 5'-AAT TCA GAG GAA GGT CC-3', anti-sense: 5'-ATG TGA CAT CTC CAT CCA GT-3') with 36 cycles (95°C for 40 s, 57 °C for 30 s and 72 °C for 60 s), N-cadherin (sense: 5'-TCA CTG CTC AGG ACC CAG AT-3', anti-sense: 5'-TAA GCC GAG TGA TGG TCC AA-3'), Vimentin (sense: 5'-GGA CCA GCT AAC CAA CGA CA-3', anti-sense: 5'-AAG GTC AAG ACG TGC CAG AG-3'), Slug (SNAI2) (sense: 5'-ACG CCT CCA AAA AGC CAA AC-3', anti-sense: 5'-ACT CAC TCG CCC CAA AGA TG-3'), E-cadherin (sense: 5'- CCG GAA AAT GAA AAA GGC CC-3', anti-sense: 5'- GGA TCT TGG CTG AGG ATG GT-3'), Catalase (sense: 5'- AGG GTG GTG CTC CAA ATT AC-3', anti-sense: 5'- TTG AAT CTC CGC ACT TCT CC-3'), SOD1 (sense: 5'- GCA AAG GTG GAA ATG AAG A-3', anti-sense: 5'-TAG CAG GAT AAC AGA TGA GTT-3'), SOD2 (sense: 5'- CAA GCC TGG TAC ATA CTG A-3', anti-sense: 5'-TTT GAT GGT TGA CAG ATT CTT T-3'), HMOX1 (sense: 5'-AAG ACT GCG TTC CTG CTC AA-3', anti-sense: 5'-AAA GTT CAT GGC CCT GGG AG-3'), BAFF-R (sense: 5'-AGA AGC ACA GAC ACT ACA-3', anti-sense: 5'-GTC AAA GAT GGT GAG GTC-3'), TACI (sense: 5'-AGC AAG GCA AGT TCT ATG A-3', anti-sense: 5'-CTC TGG TGG AAG GTT CAC-3'), BCMA (sense: 5'-AAG CAG GCG AAG TTC ATT-3', anti-sense: 5'-GGA AGG ACA AGT AAT ATC TCT ACA-3') and β-actin (sense: 5'-GCC AGG TCA TCA CCA TTG-3', anti-sense: 5'-GTT GAA GGT AGT TTC GTG GAT-3'). The PCR products were examined using agarose gel electrophoresis. Amplified PCR products were separated by 1.0 ~ 1.5% agarose gel electrophoresis and detected on Ugenius 3^®^ gel documentation system (Syngene, Cambridge, United Kingdom) 52.

### Chromatin immunoprecipitation (ChIP) assay

ChIP assay with Poly (I:C)-treated Beas-2B cells was performed by the method reported previously 53, 54. Cells were crosslinked with final concentration 1 % formaldehyde for 10 min at room temperature. Then, 125 mM glycine was added to quench unreacted formaldehyde. Cells were gathered and sonicated to make DNA fragments with a size range of 200 to 1,000 bp. Cell extracts were immune-precipitated using 1 μg anti-Nrf2 or rabbit IgG control (Abcam, Cambridge, UK) for each sample suspended in 450 μL ChIP dilution buffer (0.01% SDS, 1.1% Triton X-100, 1.2 mM EDTA, 16.7 mM Tris-HCl, pH 8.1, 167 mM NaCl). Then, samples were incubated with Protein A/G- agarose bead (P9203-015) purchased from GenDEPOT (Baker, TX, USA) to collect immunoglobulin-bound DNA fragments. Then, DNA was eluted with elution buffer (1% SDS, 100 mM NaHCO_3_) and PCR analysis were performed by using primer sets spanning Nrf2 binding site on BAFF promoter. Primers for ChIP assay were obtained from BAFF promoter sequence reported previously 4, 5. Sequences for primer set were 5'- ATT AAT TAT TTT TAT GAC AGC -3' (sense) and 5'- GTT TTT GTA AGA ATT TCA -3' (anti-sense), which corresponds to -500 to -251 bp on BAFF promoter.

### Cytoplasmic and nuclear fraction extraction

Cells were treated with Poly (I:C) for appropriate time. Cell pellet was collected by centrifugation and resuspended in hypotonic lysis buffer (HLB, 10 mM Tris·Cl, pH 7.5, 10 mM KCl, 3 mM MgCl_2_, 1 mM DTT, 0.3 % (v/v) NP-40 and 10 % (v/v) Glycerol). Cells were incubated on ice for 10 min and lysed by gentle pipetting. After centrifugation at 200 ×g for 2 min, supernatant was collected as cytosol fraction. The remaining pellet was washed with nuclear lysis buffer (NLB, 20 mM Tris·Cl, pH 7.5, 400 mM NaCl, 3 mM MgCl_2_, 1 mM DTT, 0.3 % (v/v) NP-40 and 10 % (v/v) Glycerol) to eliminate cytosol residue. Then, the pellet was resuspended in NLB, subjected to vortex briefly, pipetting several times and centrifuged at 14,000 ×g for 15 min. Supernatant was collected as nuclear fraction.

### Western blot analysis

Cells were lysed in ice-cold RIPA buffer (1% Triton X-100,) containing protease (2 μg/ml aprotinin, 1 μM pepstatin, 1 μg/ml leupeptin, 1 mM phenylmetylsufonyl fluoride (PMSF), 5 mM sodium fluoride (NaF) and 1mM sodium orthovanadate (Na_3_VO_4_)). The protein concentration of the sample was measured using SMART^TM^ BCA protein assay kit (Pierce 23228) from iNtRON Biotech. Inc. (Seoul, Rep. of Korea). Same amount of heat-denatured protein in sodium dodecyl sulfate (SDS) sample buffer was separated in sodium dodecyl sulfate polyacrylamide gel electrophoresis (SDS-PAGE), and then transferred to nitrocellulose membrane by using electro blotter. Equal amount of loaded sample on membrane was verified by ponceau S staining. The membrane was incubated with blocking solution (5% non-fat skim milk in Tris-buffered saline with Tween 20 (TBST)), and then followed by incubation with the specific primary antibodies. Horse radish peroxidase (HRP)- or infrared (IR) fluorescence dye -conjugated secondary antibody were used for target-specific primary antibody. Immuno-reactive target bands were visualized by the reaction with enhanced chemiluminescence (ECL-PS250) (Dongin LS, Seoul, Rep. of Korea) on X-ray film (Agfa HealthCare, Seol, Rep. of Korea) or by the detection of IRdye with Odyssey CLx Infrared Imaging System (LI-COR Biosciences, Lincoln, NE, Germany), respectively 52.

### Statistical analysis

Experimental differences were verified for statistical significance using ANOVA and student's t-test. P value of < 0.05 and < 0.01 was considered to be significant.

## Results

### Poly (I:C) increased Beas-2B cell migration and an expression of BAFF and EMT-associated gene

**Figure 1:**
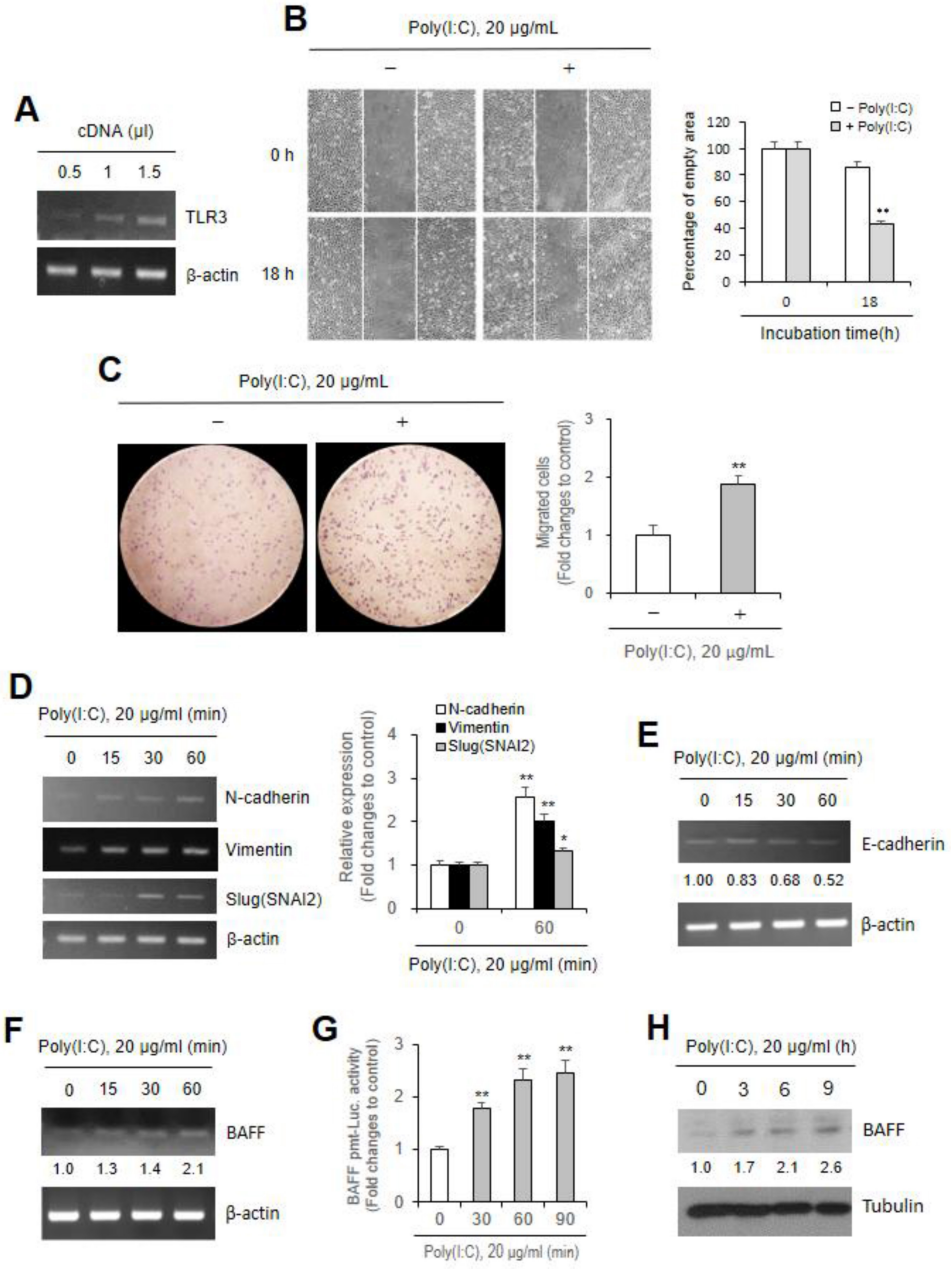
Poly (I:C) treatment enhanced Beas-2B cell migration with an increased expression of N-cadherin, Vimentin, Slug (SNAI2) and BAFF gene. **(A)** Total RNA was extracted from Beas-2B cells by using NucleoZOL reagent and TLR3 transcripts in various amount of cDNA were detected by PCR. **(B, C)** Beas-2B cells were plated on 35-mm^2^ dishes and incubated for 24 h. A confluent monolayer of Beas-2B cells was then scratched with a sterile pipette tip, treated with 20 μg/ml Poly (I:C) and incubated for 18 h. Migration of cells into the space left by the scratch was photographed using a phase-contrast microscope at 200× magnification (B left). Empty area remained in scratched region at each time point was analyzed by using ImageJ analysis software (version 1.34n). Percentage of empty area remained in scratched region that was inversely correlated with cell migration was presented as bar graph (B right). 15,000 cells in FBS-deficient media were plated to 'insert' and allowed to migrate in the absence or presence of 20 μg/ml poly (I:C) for 12 h. Then, cells were fixed, stained, and washed with water to remove redundant staining. Cells migrated underneath 'insert' membrane were photographed (C left), counted and presented as bar graph after cells on upper part of 'insert' membrane were wiped out (C right). **(D-G)** Beas-2B cells were incubated with 20 μg/ml poly (I:C) for 15-60 min. Total RNA was extracted from Beas-2B cells by using NucleoZol reagent. Transcripts of N-cadherin, Vimentin, Slug (SNAI2) (D left), E-cadherin (E) and BAFF (F) were detected by PCR. Beas-2B cells were transfected with pGL3-BAFF-promter (pmt) plasmid, incubated, re-plated and treated with 20 μg/ml poly (I:C) for 30-90 min. Cell lysates were prepared and luciferase activity was measured by using luminometer followed by the incubation with substrate, luciferin. Fold changes relative to the control were presented as bar graph (G). **(H)** Beas-2B cells were incubated with 20 μg/ml poly (I:C) for 3-9 h. Cell lysates from each culture dish were prepared for the detection of target protein, BAFF, by western blot analysis. Relative intensities of each band were normalized by comparison to actin (D left, E, F) or to tubulin (H) followed by a quantitation with ImageJ (version 1.34n). Fold changes relative to the control were presented as bar graph (D right) or indicated as number under each band (E, F, H). Each experiment was performed at least four times. Data in bar graphs are represented as means ± SD and were statistically analyzed by using ANOVA and student's t-test. **p<0.01, significantly different from poly (I:C)-untreated group (B-D right, G).

Previous studies have reported an effect of TLR3 and the TLR3 agonist Poly (I:C) on epithelial damage in the skin and heart 22-25, we examined TLR3 expression in Beas-2B cells (Fig. 1A). As bronchial epithelial cell migration plays a pivotal role in the airway repair and remodeling that occurs in respiratory diseases 15, we therefore examined the effects of Poly (I:C) on Beas-2B cell migration. No differences in Beas-2B cell viability were detected in response to the tested concentrations of Poly (I:C), as measured by trypan blue exclusion assay (data not shown). Poly (I:C) significantly increased cell migration ≈58% at 18 h, compared to ≈13% in control group (Fig. 1B). Increased cell migration was confirmed by using Transwell migration assay. Cell migration by Poly (I:C)-treated cells was ≈2.0 fold higher than in control cells (Fig. 1C). These results were associated with the expression of N-cadherin, Vimentin or Slug (SNAI2) gene increased ≈2.6, ≈2.0, and ≈1.3 fold, respectively by Poly (I:C) treatment for 60 min (Fig. 1D). Increased expressions of those three molecules are reflected in a decrease in E-cadherin transcript levels, which were lowered to approximately 0.83, 0.68 and 0.52 fold of Poly (I:C)-untreated control level following Poly (I:C) treatment for 15, 30, and 60 min, respectively (Fig. 1E). Since BAFF mRNA in airway epithelial cells was significantly up-regulated by Poly (I:C) 9, we also determined whether BAFF expression is enhanced by Poly (I:C) under our experimental condition. Data showed an increased level in BAFF transcript, promoter activity and protein. BAFF transcript level was ≈1.3, ≈1.4 and ≈2.1 fold higher by Poly (I:C) treatment for 15, 30, and 60 min compared to Poly (I:C)-untreated control (Fig. 1F). BAFF promoter activity was increased ≈1.8, ≈2.3 and ≈2.5 fold by Poly (I:C) treatment for 30, 60, and 90 min compared to Poly (I:C)-untreated control (Fig. 1G). BAFF protein level was ≈1.7, ≈2.1 and ≈2.6 fold higher by Poly (I:C) treatment for 3, 6, and 9 h compared to Poly (I:C)-untreated control (Fig. 1H). These results suggest that Poly (I:C)-induced cell migration is associated with BAFF expression. Therefore, our subsequent research will focus on elucidating the relationship between BAFF expression and the induction of the above three EMT markers rather than their correlation with E-cadherin downregulation.

### Beas-2B cell migration by Poly (I:C) treatment was attenuated by siBAFF

**Figure 2:**
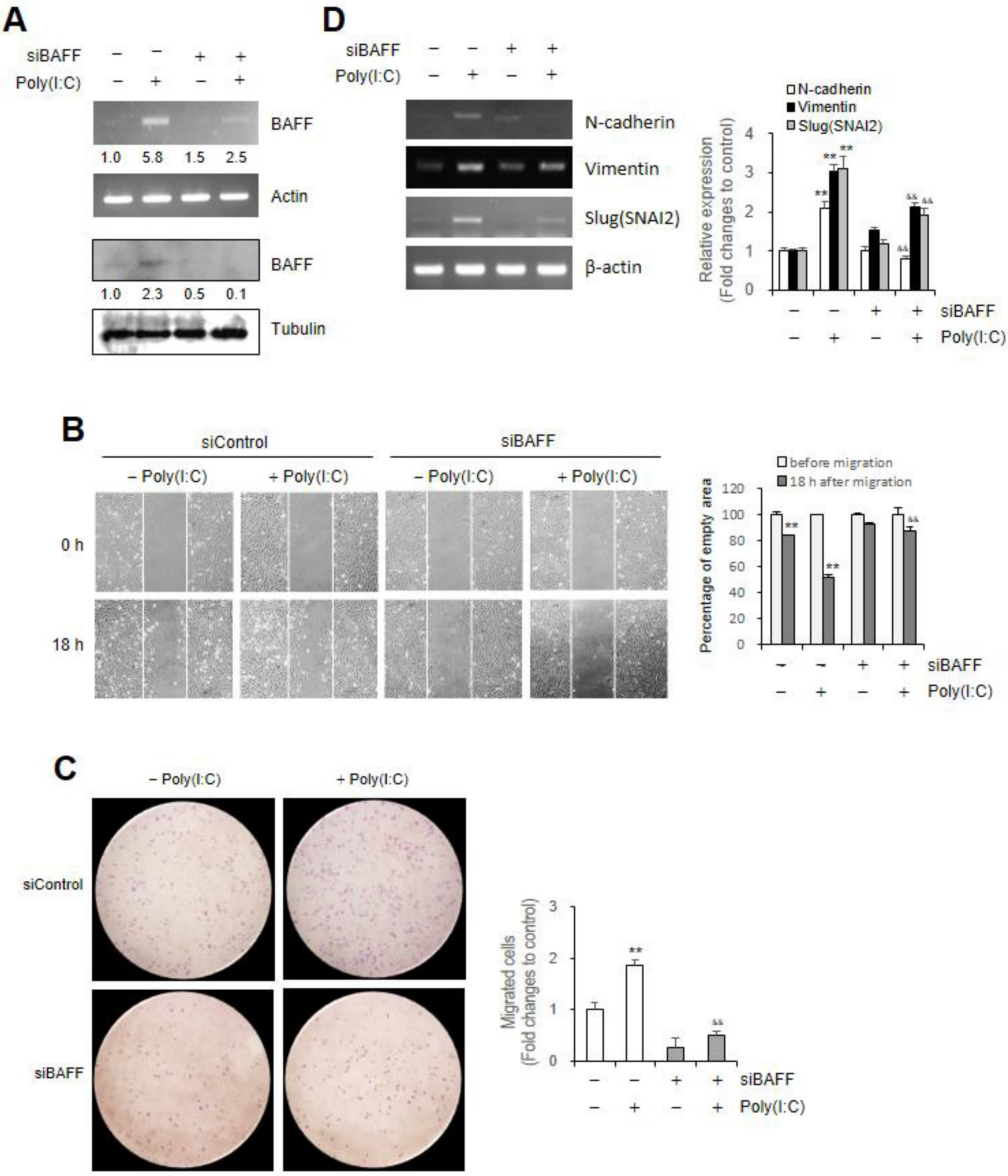
Beas-2B cell migration by Poly (I:C) treatment was attenuated by small interference (si) RNA of BAFF. **(A-D)** Beas-2B cells were transfected with control siRNA (siControl) or BAFF-siRNA (siBAFF). Then, cells were incubated in the absence or presence of 20 μg/ml Poly (I:C). Total RNA was extracted from Beas-2B cells by using NucleoZOL reagent. Transcripts of BAFF (A top), N-cadherin, Vimentin, Slug (SNAI2) (B left) were detected by PCR. Cell lysates from each culture dish were prepared for the detection of target protein, BAFF, by western blot analysis (A bottom). Relative intensities of each band were normalized by comparison to actin (A top, D) or tubulin (A bottom) followed by a quantitation with ImageJ (version 1.34n). Fold changes relative to the control were indicated as number under each band (A) or presented as bar graph (D right). Beas-2B cells from siControl- or siBAFF-transfected group were plated on 35-mm^2^ dishes and incubated for 24 h. A confluent monolayer of Beas-2B cells was then scratched with a sterile pipette tip, treated with 20 μg/ml Poly (I:C) and incubated for 18 h. Migration of cells into the space left by the scratch was photographed using a phase-contrast microscope at 200× magnification (B left). Empty area remained in scratched region at each time point was analyzed by using ImageJ analysis software (version 1.34n). Percentage of empty area remained in scratched region that was inversely correlated with cell migration was presented as bar graph (B right). 15,000 siControl- or siBAFF-transfected cells in FBS-deficient media were plated to 'insert' and allowed to migrate in the absence or presence of 20 μg/ml poly (I:C) for 12 h. Then, cells were fixed, stained, and washed with water to remove redundant staining. Cells migrated underneath 'insert' membrane were photographed (C left), counted and presented as bar graph after cells on upper part of 'insert' membrane were wiped out (C right). Each experiment was performed at least four times. Data in bar graphs are represented as means ± SD and were statistically analyzed by using ANOVA and student's t-test. **p<0.01, significantly different from Poly (I:C)-untreated group. ^&&^*p*<0.01, significantly different from Poly (I:C)-treated and siBAFF-untreated group (B-D right).

To examine the effect of BAFF in Poly (I:C)-induced Beas-2B cell migration, we transfected cells with small interference (si) RNA of BAFF gene to inhibit BAFF expression. BAFF transcript and protein level increased by Poly (I:C) ≈5.8 and ≈2.3 fold, which was respectively reduced to ≈2.5 (Fig. 2A top) and ≈0.1 (Fig. 2A bottom) fold in siBAFF-transfected group compared to control. Beas-2B cells were treated with Poly (I:C) in the absence or presence of BAFF-siRNA. Under the latter condition, Poly (I:C)-mediated cell migration was attenuated (Fig. 2B left), as evidenced by ≈49% migration after 18 h in control cells but only ≈12% in siBAFF-treated group (Fig. 2B right). Poly (I:C)-induced Beas-2B cell migration was further examined by Transwell migration assay (Fig. 2C left). Cell migration was reduced from ≈1.9 fold in Poly (I:C)-treated cells to ≈0.6 fold in group treated with Poly (I:C) and BAFF-siRNA compared to control (Fig. 2C right). These results were associated with decrease in N-cadherin, Vimentin or Slug (SNAI2) gene expression increased by Poly (I:C) treatment from ≈2.1, ≈3.0, and ≈3.1 fold to ≈0.8, ≈2.1, and ≈1.9 fold in siBAFF-transfected group compared to control untreated with both, respectively (Fig. 2D). It demonstrates that Poly (I:C)-induced Beas-2B cell migration is associated with BAFF expression.

### JNK phosphorylation by Poly (I:C) regulates BAFF expression and cell migration

**Figure 3:**
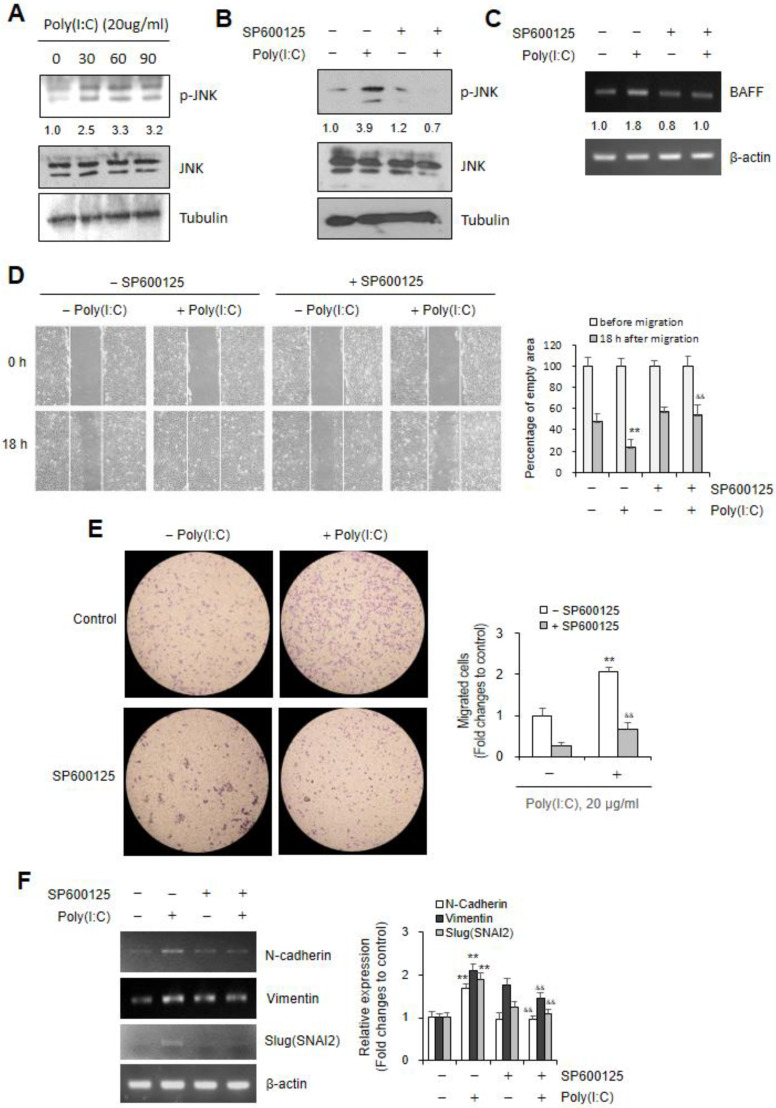
Beas-2B cell migration by Poly (I:C) treatment was dependent on JNK phosphorylation (p)-mediated BAFF expression. **(A-F)** Beas-2B cells were treated with 20 μg/ml Poly (I:C) in the absence (A-G) or presence (B-G) of SP600125, JNK inhibitor. Cell lysates from each culture dish were prepared for the detection of target proteins, JNK or p-JNK by western blot analysis (A, B). Total RNA was extracted from each cultured Beas-2B cells by using NucleoZOL reagent. Transcripts of BAFF (C), N-cadherin, Vimentin, Slug (SNAI2) (F left) were detected by PCR. Relative intensities of each band were normalized by comparison to JNK and tubulin (A, B) or actin (C. F left) followed by a quantitation with ImageJ (version 1.34n). Fold changes relative to the control were indicated as number under each band (A-C) or presented as bar graph (F right). A confluent monolayer of Beas-2B cells plated on 35-mm^2^ dishes was scratched with a sterile pipette tip and incubated for 18 h. Migration of cells into the space left by the scratch was photographed using a phase-contrast microscope at 200× magnification (D left). Empty area remained in scratched region at each time point was analyzed by using ImageJ analysis software (version 1.34n). Percentage of empty area remained in scratched region that was inversely correlated with cell migration was presented as bar graph (D right). 15,000 cells plated to 'insert' in FBS-deficient media were allowed to migrate for 12 h. Then, cells were fixed, stained, and washed with water to remove redundant staining. Cells migrated underneath 'insert' membrane were photographed (E left), counted and presented as bar graph after cells on upper part of 'insert' membrane were wiped out (E right). Each experiment was performed at least four times. Data in bar graphs are represented as means ± SD and were statistically analyzed by using ANOVA and student's t-test. **p<0.01, significantly different from Poly (I:C)-untreated group. ^&&^*p*<0.01, significantly different from Poly (I:C)-treated and SP600125-untreated group (D-F right).

As Poly (I:C) increased the phosphorylation of various kinase in microglial cells 26, we investigated the mechanism of signaling pathway to regulate Poly (I:C)-mediated BAFF expression and cell migration. In cells treated with Poly (I:C) for 30 ~ 90 min, JNK phosphorylation was approximately ≈2.5 ~ ≈3.3 fold higher than in control cells (Fig. 3A). As SP600125, JNK inhibitor, exhibits comparable inhibitory effects on JNK-mediated cell functions 55, we treated cells with SP600125 to confirm the effects of poly (I:C) on JNK phosphorylation. Beas-2B cells were incubated with Poly (I:C) in absence or presence of SP600125. JNK phoshorylation was significantly decreased from ≈3.9 fold in Poly (I:C)-treated cultures to ≈0.7 fold in cultures co-treated with Poly (I:C) and SP600125 compared to control group (Fig. 3B). BAFF transcript level was significantly decreased from ≈1.8 fold in Poly (I:C)-treated cultures to ≈1.0 fold in cultures co-treated with Poly (I:C) and SP600125 compared to control group (Fig. 3C). To examine the role of JNK on airway epithelial cell migration, Beas-2B cells were treated with Poly (I:C) in the absence or presence of SP600125. Under the latter condition, Poly (I:C)-mediated cell migration was attenuated (Fig. 3D left), as evidenced by ≈78% migration after 18 h in these cells but only ≈45% in SP600125-treated group (Fig. 3D right). Poly (I:C)-induced Beas-2B cell migration was further examined by Transwell migration assay (Fig. 3E left). Cell migration was reduced from ≈2.1 fold in Poly (I:C)-treated cells to ≈0.6 fold in group co-treated with Poly (I:C) and SP600125 compared to control (Fig. 3E right). These results were associated with decrease in N-cadherin, Vimentin or Slug (SNAI2) gene expression increased ≈1.7, ≈2.1, and ≈1.9 fold by Poly (I:C) treatment to ≈0.9, ≈1.5, and ≈1.1 fold in group co-treated with Poly (I:C) and SP600125, compared to control group untreated with both, respectively (Fig. 3F). These results demonstrate that Poly (I:C)-induced Beas-2B cell migration is associated with JNK phosphorylation-related BAFF expression.

### Poly (I:C) increased ROS production leading to JNK-associated Nrf2 activation in Beas-2B cells

**Figure 4:**
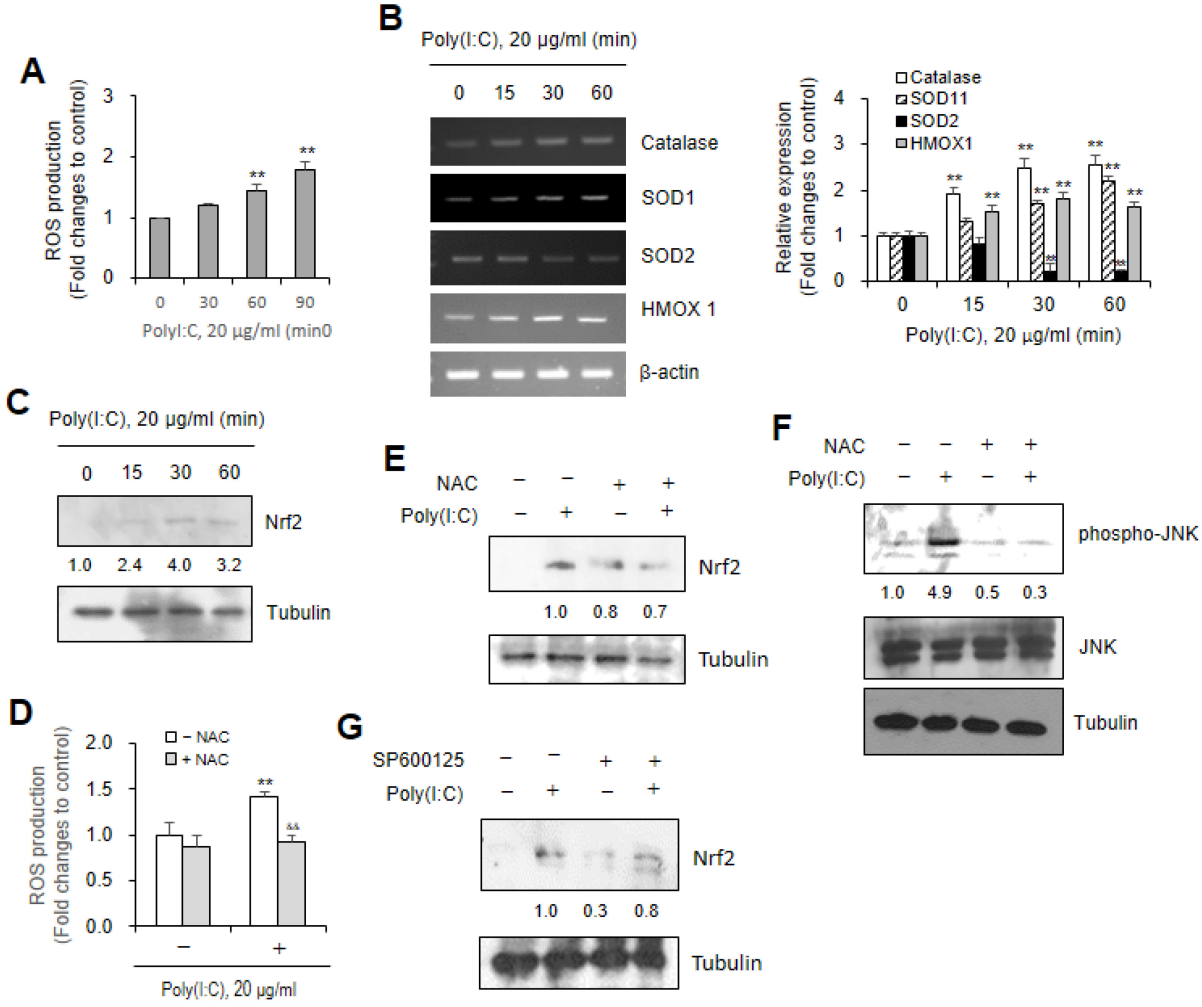
Poly (I:C) treatment increased ROS production leading to JNK-associated Nrf2 activation in Beas-2B cells. **(A-G)** Beas-2B cells were treated with 20 μg/ml Poly (I:C) in the absence (A-G) or presence of N-acetylcysteine (NAC) (D-F) or SP600125 (G). ROS production was assessed by the measurement of fluorescence intensity changes with 2',7'-dichlorodihydrofluorescein diacetate (DCF-DA) (A, D). Total RNA was extracted from each cultured Beas-2B cells by using NucleoZOL reagent. Transcripts of Catalase, SOD1, SOD2 and HMOX1 were detected by PCR (B left). Cell lysates from each culture dish were prepared for the detection of target proteins, Nrf2 (C, E, G), JNK or p-JNK (F) by western blot analysis. Relative intensities of each band were normalized by comparison to actin (B left), to tubulin (C, E, G) or JNK and tubulin (F) followed by a quantitation with ImageJ (version 1.34n). Fold changes relative to the control were presented as bar graph (B right) or indicated as number under each band (C, E, F, G). Each experiment was performed at least four times. Data in bar graphs are represented as means ± SD and were statistically analyzed by using ANOVA and student's t-test. **p<0.01, significantly different from Poly (I:C)-untreated group (A, B right, D). ^&&^*p*<0.01, significantly different from Poly (I:C)-treated and NAC-untreated group (D).

As ROS activate multiple signaling pathways, including MAPK pathways like p38, JNK, and ERK 41, we investigated the role of ROS to regulate Poly (I:C)-mediated BAFF expression and cell migration. ROS production was time-dependently increased ≈1.2 ~ ≈1.8 fold higher in cells treated with Poly (I:C)/mL for 30 ~ 90 min than in control cells (Fig. 4A). In addition, antioxidant expression was increased by the treatment with Poly (I:C) for 15 ~ 60 min. Except SOD2, Catalase expression increased ≈1.9 ~ ≈2.6 fold, SOD1 ≈1.3 ~ ≈2.2 fold, HMOX1 ≈1.5 ~ ≈1.8 fold higher in Poly (I:C)-treated cells than in control cells (Fig. 4B). As ROS lead to Nrf2 activation 41, 42, we investigated whether Poly (I:C)-induced ROS are associated with not only Nrf2 activation but also JNK phosphorylation. Poly (I:C) treatment for 15, 30 and 60 min increased Nrf2 ≈2.4, ≈4.0 and ≈3.2 fold, respectively (Fig. 4C).

Since NAC, as an antioxidant has been widely used to reduce oxidative stress 39, 56, we used NAC to examine the effects of ROS level induced by Poly (I:C) treatment. ROS production increased ≈1.4 fold by Poly (I:C) treatment were inhibited to ≈0.9 fold in response to co-treatment with NAC (Fig. 4D). Nrf2 protein amount increased by Poly (I:C) treatment were inhibited to ≈0.7 fold (Fig. 4E) and JNK phosphorylation from ≈4.9 to ≈0.3 fold (Fig. 4F) in response to co-treatment with NAC. In addition, Nrf2 protein amount increased by Poly (I:C) treatment were inhibited to ≈0.8 fold in response to co-treatment with SP600125 (Fig. 4G). These results suggest that Poly (I:C)-induced ROS production regulate Nrf2 activation via JNK phosphorylation in Beas-2B cells.

### Poly (I:C)-induced ROS augmented BAFF expression via Nrf2 binding on BAFF promoter

**Figure 5:**
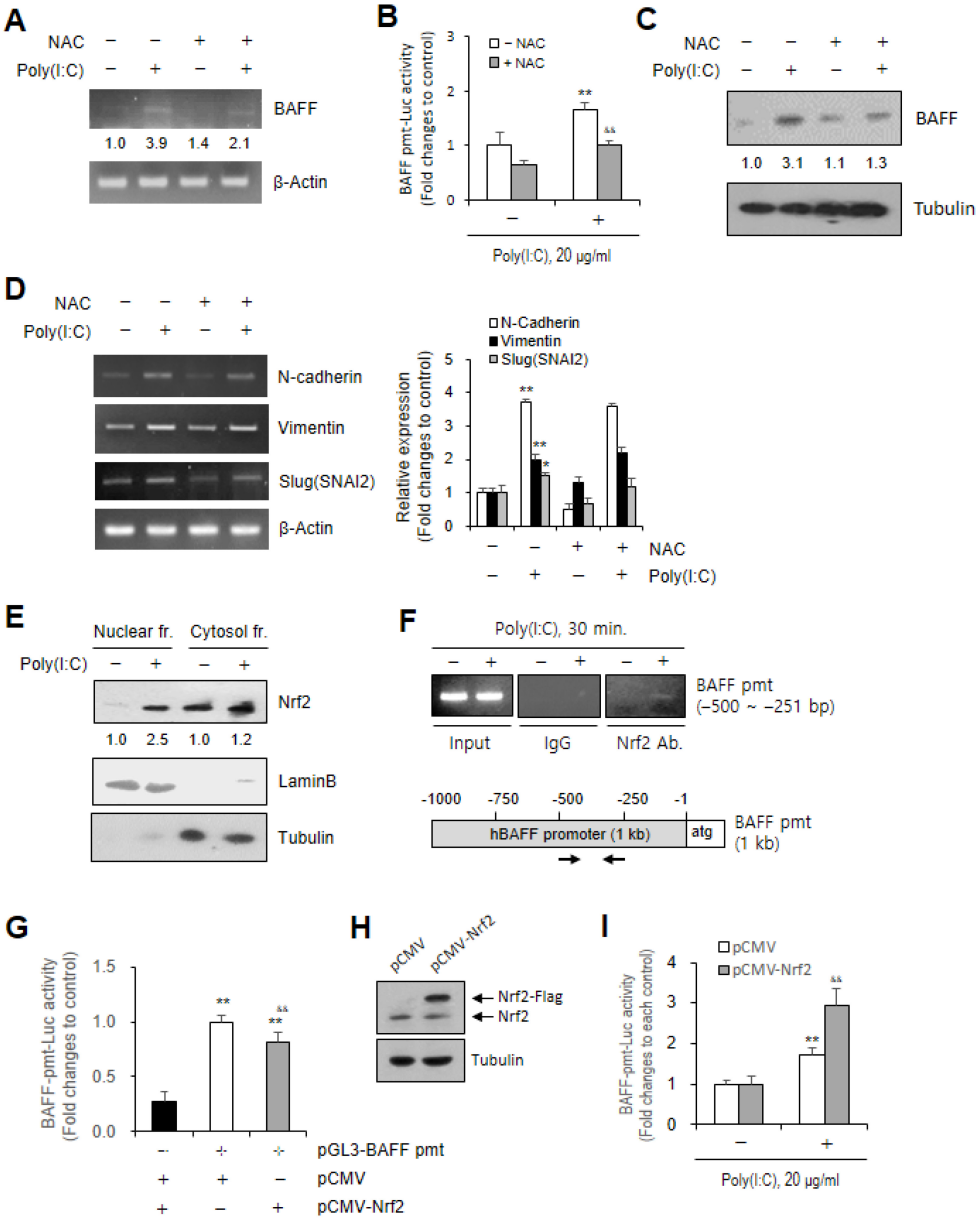
Poly (I:C)-induced BAFF expression was associated with ROS production and Nrf2 binding on BAFF promoter. **(A-F)** Beas-2B cells were treated with 20 μg/ml Poly (I:C) in the absence (A-F) or presence (A-D) of N-acetylcysteine (NAC). Total RNA was extracted from each cultured Beas-2B cells by using NucleoZOL reagent. Transcripts of BAFF (A), N-cadherin, Vimentin, Slug (SNAI2) (D left) were detected by PCR. Beas-2B cells were transfected with pGL3-BAFF-promter (pmt) plasmid, incubated, re-plated and treated with 20 μg/ml poly (I:C). Cell lysates were prepared and luciferase activity was measured by using luminometer followed by the incubation with substrate, luciferin. Fold changes relative to the control were presented as bar graph (B). Cell lysates from each culture dish were prepared for the detection of target protein, BAFF by western blot analysis (C). Cytosol and nuclear fraction were separated for the detection of target protein, BAFF by western blot analysis (E). Relative intensities of each band were normalized by comparison to actin (A, D left), to tubulin (C, E) or to laminB (E) followed by a quantitation with ImageJ (version 1.34n). Fold changes relative to the control were indicated as number under each band (A, C, E) or presented as bar graph (D right). Beas-2B cells were fixed with 10 % formaldehyde. Their chromatin extracts were immunoprecipitated with anti-Nrf2 antibodies. DNA fragments were subjected to PCR analysis using primer sets spanning the promoter regions. Sequences for primer set were 5'-ATT AAT TAT TTT TAT GAC AGC-3' (sense) and 5'-GTT TTT GTA AGA ATT TCA-3' (anti-sense) corresponding to -500 to -251 bp on BAFF promoter (F). **(G-I)** Beas-2B cells were co-transfected with pGL3-BAFF-promter (pmt) and pCMV or pCMV-Nrf2 plasmids (G, I) and treated with 20 μg/ml poly (I:C) (I). Cell lysates were prepared and luciferase activity was measured by using luminometer followed by the incubation with substrate, luciferin. Fold changes relative to each control were presented as bar graph (G, I). Cell lysates were prepared and expressed protein was detected by western blot analysis (H). Each experiment was performed at least four times. Data in bar graphs are represented as means ± SD and were statistically analyzed by using ANOVA and student's t-test. **p<0.01, significantly different from poly (I:C)-untreated group (B, D right), pGL3-BAFF-pmt plasmid-untransfected group (G) or poly (I:C)-untreated group and pCMV plasmid-transfected group (I). ^&&^*p*<0.01, significantly different from Poly (I:C)-treated and NAC-untreated group (B, D right), pGL3-BAFF-pmt plasmid- and pCMV plasmid-transfected group (G) or pCMV plasmid-transfected and Poly (I:C)-treated group (I).

To examine the effect of ROS on BAFF expression, we used materials, NAC or Nrf2 plasmid and method, chromatin immunoprecipitation (ChIP). BAFF transcripts increased ≈3.9 fold by Poly (I:C) treatment were decreased to ≈2.1 fold (Fig. 5A), BAFF transcriptional activity ≈3.9 to ≈1.0 fold (Fig. 5B), BAFF protein amount ≈3.1 to ≈1.3 fold (Fig. 5C) by co-treatment Poly (I:C) with NAC. In contrast, no or a little change in N-cadherin, Vimentin or Slug (SNAI2) gene expression was observed by co-treatment with NAC (Fig. 5D). Results demonstrate that ROS production could be applied to regulate BAFF expression but not N-cadherin, Vimentin or Slug (SNAI2) genes. ROS-mediated BAFF gene expression was confirmed by Nrf2 binding on BAFF gene promoter. We observed nuclear localization of Nrf2 ≈2.5 fold higher in Poly (I:C)-treated cells than in control (Fig. 5E). Then, it is assessed whether nuclear Nrf2 bind on BAFF gene promoter by using ChIP assay following the analysis of Nrf2 binding sites on BAFF promoter with JASPAR, an open-access online database of transcription factor binding profiles. We found that Nrf2 bind on upstream -500 ~ -251 bp of BAFF gene promoter. When promoters of three EMT markers were also analyzed by JASPAR, Nrf2 binding sites were expected in N-cadherin and Slug (SNAI2) promoters but not in Vimentin. However, no Nrf2 binding on N-cadherin and Slug (SNAI2) promoters was detected by ChIP assay either (data not shown). We confirmed the effect of Nrf2 on BAFF gene promoter by determining transcriptional activity of BAFF gene promoter. When Beas-2B cells were co-transfected with BAFF promoter and Nrf2 plasmids, the transcriptional activity of BAFF gene promoter was reduced in co-transfected cells compared to control group untransfected with Nrf2 plasmid (Fig. 5G). Flag-tagged Nrf2 band in Nrf2 plasmid-transfected cells was upper to endogenous Nrf2, which was detected by western blot analysis (Fig. 5H). When the cells co-transfected with BAFF promoter and Nrf2 plasmids were treated with Poly (I:C), the transcriptional activity of BAFF gene promoter increased ≈2.9 fold in co-transfected cells, which was higher compared to ≈1.7 fold in control group untransfected with Nrf2 plasmid. These data demonstrate that Poly (I:C)-induced BAFF expression was dependent on activation and nuclear localization of Nrf2 to bind on BAFF gene promoter.

### BAFF protein increased Beas-2B cell migration and EMT-associated gene expression

**Figure 6:**
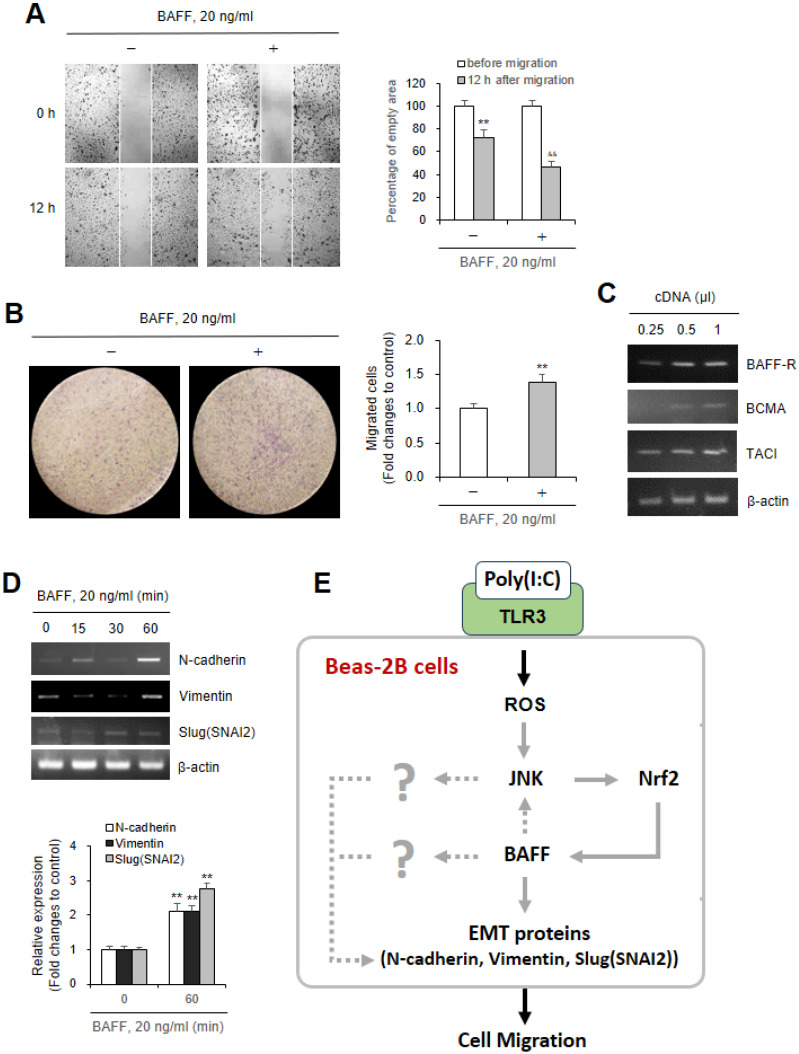
Beas-2B cell migration was dependent on BAFF protein produced by poly (I:C) treatment. **(A, B)** Beas-2B cells were plated on 35-mm^2^ dishes and incubated for 24 h. A confluent monolayer of Beas-2B cells was then scratched with a sterile pipette tip, treated with 20 ng/ml BAFF protein and incubated for 12 h. Migration of cells into the space left by the scratch was photographed using a phase-contrast microscope at 200× magnification (A left). Empty area remained in scratched region at each time point was analyzed by using ImageJ analysis software (version 1.34n). Percentage of empty area remained in scratched region that was inversely correlated with cell migration was presented as bar graph (A right). 15,000 cells in FBS-deficient media were plated to 'insert' and allowed to migrate in the absence or presence of 20 ng/ml BAFF protein for 6 h. Then, cells were fixed, stained, and washed with water to remove redundant staining. Cells migrated underneath 'insert' membrane were photographed (B left), counted and presented as bar graph after cells on upper part of 'insert' membrane were wiped out (B right). **(C, D)** Total RNA was extracted from Beas-2B cells untreated or treated with 20 ng/ml BAFF protein by using NucleoZOL reagent. Transcripts of BAFF-R, TACI and BCMA were detected by PCR (C). Transcripts of N-cadherin, Vimentin, Slug (SNAI2) were detected by PCR (D top). Relative intensities of each band were normalized by comparison to actin followed by a quantitation with ImageJ (version 1.34n). Fold changes relative to the control were presented as bar graph (D bottom). Each experiment was performed at least four times. Data in bar graphs are represented as means ± SD and were statistically analyzed by using ANOVA and student's t-test. **(E)** Scheme about action mechanism of Poly (I:C) on airway epithelial cell migration by ROS production. Airway epithelial cell migration is regulated by BAFF expression through intracellular ROS-JNK-Nrf2 axis (grey solid lines) after Poly (I:C) bind to TLR3. BAFF enhanced cell migration through an increase in EMT proteins, N-cadherin, Vimentin, Slug (SNAI2) (grey solid lines) or by some signaling factors (indicated as question mark), directly regulated or indirectly via JNK activation or BAFF-independent pathway (grey dotted lines). Our findings are represented by grey solid lines.

To confirm that airway epithelial cell migration by Poly (I:C) treatment was associated with BAFF production, Beas-2B cells were incubated with BAFF protein. As shown in Fig. 6A and B, BAFF protein significantly increased cell migration ≈54% at 12 h, compared to ≈28% in control group (Fig. 6A). Increased cell migration was confirmed by using Transwell migration assay. Cell migration by BAFF protein-treated cells was ≈1.4 fold higher than in control cells (Fig. 6B). As we observed the expression of three BAFF receptors, BAFF-R, TACI and BCMA on Beas-2B cells (Fig. 6C), we examined the expression of three EMT molecules by the treatment with BAFF protein. These results were associated with the expression of N-cadherin, Vimentin or Slug (SNAI2) gene increased ≈2.1, ≈2.7, and ≈2.1 fold by the treatment with BAFF protein for 60 min, respectively (Fig. 6D). Our data showed that These data suggest that Poly (I:C)-induced BAFF expression enhances the regenerative migration of airway epithelial cells mediated by EMT molecules like N-cadherin, Vimentin or Slug (SNAI2) gene (Fig. 6E).

## Discussion

BAFF is produced by different types of extracellular molecules including Poly (I:C) from immune cells 5, 6 or non-immune cells 3, 4, 7, 8 including airway cells 9, 21. BAFF regulates various B cell functions 1, 2. However, it may be unknown about the role of BAFF in other cells like airway cells in epithelium, which act not only as a physical barrier but also modulate immune responses 11-13. Airway epithelial cells also affect airway repair and remodeling by local environmental changes 14-16 such as EMT to increase cellular plasticity and to replace damaged specialized cells in organs 33. EMT is characterized by decreased E-cadherin and increased expression in N-cadherin, Vimentin and fibronectin 37. However, it has not been known about whether BAFF in Poly (I:C)-treated airway cells plays a potential role in EMT. Understanding the mechanisms underlying BAFF induction and function in response to Poly (I:C) is critical for elucidating the complex interactions between epithelial cells and the immune system in airway diseases. Here, we investigated the role of TLR3-mediated signaling to stimulate airway cell migration through BAFF expression by using TLR3 agonist Poly (I:C) and Beas-2B human bronchial epithelial cells. Results demonstrate that the *in vitro* incubation of Beas-2B cells with Poly (I:C) induces BAFF expression and stimulates the loss of epithelial cell integrity through ROS production and JNK/Nrf2 activation.

BAFF expression is increased in non-immune cells, synoviocytes 5, 6, human intestinal epithelial cells 7, salivary gland 8 and airway cells 9 as well as in immune cells 3, 4. Our data also indicate that the Poly (I:C)-mediated increase in airway epithelial cell migration is associated with an increase in BAFF expression, which is also related to the changes in N-cadherin, Vimentin and Slug (SNAI2) as main EMT molecules (Fig. 1 and 2).

Previous studies have reported the association of various molecules in TLR3 signaling pathways. YAP1 regulates TLR3-mediated repair and regeneration in diabetic osteoarthritis progression 57 or neonatal heart regeneration and repair 25. Activation of the TLR3/PI3K/Akt pathway ameliorates myocardial ischemia/reperfusion injury 58. Poly (I:C) increases Hsp27, Hsp70, and Bcl2 levels but reduces the Bax level in the treatment of ischemic brain tissues 59. Poly (I:C) also enhances the phosphorylation of various kinases including p38 MAPK, ERK, JNK and Akt in microglial cells 26. JNK-mediated functions are decreased by using SP600125 to exhibit comparable inhibitory effects 55, we also found the JNK in Beas-2B cells is phosphorylated in response to Poly (I:C), which is inhibited by SP600125 treatment, leading to the inhibition of cell migration (Fig. 3). It suggests that JNK can be a main signaling molecule to contribute Poly (I:C)-induced airway cell migration.

As ROS are reactive molecules that play critical roles in cellular signaling and homeostasis 39, 40 by the activation of multiple signaling pathways leading to Nrf2 activation and antioxidant gene expression eventually 41, 42. NAC is widely used to reduce oxidative stress by replenishing intracellular levels of antioxidants 39. Poly (I:C)-induced ROS production and Nrf2 activation were reduced by pre-incubation of Beas-2B cells with NAC (Fig. 4). Poly (I:C)-induced Nrf2 activation was also decreased by co-incubation with SP600125 (Fig. 4). We also found that NAC inhibits Poly (I:C)-mediated BAFF expression, evidenced in ChIP assay and transcriptional promoter assay as well as PCR and western blot analysis (Fig. 5). These results suggest that the mechanism linking airway cell migration and BAFF expression involves ROS production, JNK phosphorylation and therefore the potential of Nrf2 as novel mechanistic targets to increase the Poly (I:C)-induced EMT of airway epithelial cells. However, it cannot be ruled out that the results obtained using SP600125 and NAC might be due to off-target effects, rather than specific inhibition of JNK and Nrf2. Future studies should employ siJNK or siNrf2 for more precise inhibition.

EMT is stimulated by external stimuli such as growth factors, pro-inflammatory cytokines, and viral mimics like Poly (I:C) 38. We also used a recombinant human BAFF protein as an external stimulant on Beas-2B cells to better understand the role of Poly (I:C)-mediated BAFF expression on cell migration (Fig. 6). Our results demonstrate that BAFF protein treatment increases Beas-2B cell migration and the expression of EMT molecules, N-cadherin, Vimentin and Slug (SNAI2) genes (Fig. 6D). Based on the previous report that no changes were detected in total B cell number in the incubation with BAFF protein 60, BAFF primarily may affect cell migration rather than proliferation. So, our data assessed by Transwell assays and the wound healing assay may not reflect a combination of Beas-2B cell migration and proliferation. It suggests that Poly (I:C) contributes to EMT or EMT-associated migration through BAFF expression.

BAFF signaling is initiated by binding to its receptors, BAFF-R, TACI and BCMA on B cells and supports their proliferation, maturation and antibody secretion 10. Especially, BAFF in B cells act through the BAFFR receptor that crosstalk with other receptors, notably the B cell antigen receptor (BCR), leading to the interaction with TRAF3, CD79A and the activation of downstream spleen tyrosine kinase (Syk) and NF-kappaB signalling 60, 61. BAFF might attenuate the effect by oxidative stress in B cells through Syk activation 60. Syk also controls oxidative stress via nuclear translocation of Nrf2 in human B cells 62. ROS activate multiple signaling pathways, including MAPK pathways like p38 and ERK as well as JNK 41. c-Jun is the transactivation component of the heterodimeric transcription factor AP-1, along with JNK 63. ROS activate Nrf2 by disrupting the interaction between keap1 and Nrf2 41, 42. Our data showed that EMT markers are ROS-Nrf2-independent while BAFF is ROS-dependent. So, it is possible for EMT to be contributed by BAFF-independent pathways downstream of Poly (I:C) as well as BAFF-dependent pathway. Our data showed that Nrf2 binds to the BAFF promoter and regulates BAFF transcription (Fig.5F). However, no evidence is observed that Nrf2 directly regulates EMT-associated genes (data not shown). It implies a broader role for Nrf2 in EMT regulation. It suggests that Nrf2 appears to act primarily upstream of BAFF expression, while EMT marker regulation may be mediated through alternative downstream effectors of JNK signaling (Fig. 6E).

The ability of Beas-2B cells to migrate in the setting of wound repair was determined to reflect new tissue formation with the secretion of extracellular matrix proteins such as collagen, fibronectin and laminin 31, 32. We previously reported that Poly (I:C) can prevent or attenuate the airway epithelial changes induced by SARS-CoV-2 spike protein (SP) via primary cilium (PC) formation 16. It has not been determined yet about whether Poly (I:C)-induced BAFF expression or BAFF protein affect PC formation but it is possible for the regenerative effect of Poly (I:C) on SARS-CoV-2 SP to be mediated by BAFF expression. All our results suggest that Poly (I:C) may ameliorate the structural changes in the airway caused by exposure to injurious agents.

EMT is also widely associated with pathological remodeling and fibrosis as well as regenerative migration during wound repair 34-36. As cell-based results using BEAS-2B cells may represent an inducible and reversible EMT model 64, it is possible that the observed BAFF-mediated EMT represents a partial or reversible EMT state with potential pathological implications (e.g., airway remodeling), which constitutes a limitation of this study. Another limitation is that all experiments were conducted in a single immortalized bronchial epithelial cell line (BEAS-2B). While this model is widely used, it remains to be clarified whether similar mechanisms might operate in primary human airway epithelial cells or *in vivo* settings. To show whether Poly (I:C)-induced EMT and enhanced migration observed *in vitro* may relate to *in vivo* airway epithelial injury, repair processes, or viral infection, further studies are required to define the long-term *in vivo* effects of BAFF on the repair of airway epithelial damage. This research could clarify the physiological and translational relevance of our data and potentially reveal a novel regenerative mechanism.

c-Jun which is the transactivation component of the heterodimeric transcription factor AP-1, which in parallel to JNK 63. Activation of the MAPK/JNK pathway leads to the activation of transcription factors such as c-Jun, which promote the expression of EMT-related transcription factors such as Slug (SNAI2), Snail, Twist, and ZEB1 45. Transcription factors regulate EMT by binding to the gene promoters and suppress epithelial markers while promote mesenchymal genes such as N-cadherin, Vimentin, fibronectin 46, 47. So, it is possible for JNK/c-Jun activated by Poly (I:C)-mediated ROS to regulate mesenchymal genes via EMT-related transcription factor, Slug (SNAI2) but not through Nrf2. Furthermore, it is required to reveal the role of other EMT-related transcription factors and the relationship between those and Nrf2 or c-Jun.

NAC treatment markedly suppresses BAFF expression while having little effect on EMT marker expression (Fig. 5A-D). If BAFF is a central mediator linking ROS to EMT, suppression of BAFF would be expected to attenuate EMT-associated gene expression as well. However, the current results instead suggest that EMT marker induction may occur independently of BAFF, possibly through ROS-JNK-c-Jun-dependent pathways. This discrepancy should be distinguished by BAFF-dependent effects on migration from BAFF-independent regulation of EMT-associated transcription factors.

Taken together, even though all factors and the mechanism of action are not clarified, our findings indicate that ROS acts as an upstream modulator of BAFF gene expression via Nrf2 activation. ROS-mediated JNK plays as a central regulator in both BAFF regulation and EMT processes. These represent a protective feedback response against oxidative stress or alternatively, cellular shift toward a migration- promoting phenotype. Nevertheless, although we observed changes in EMT markers in Beas-2B cells, it cannot be ruled out the possibility that these results represent an EMT-associated phenotype rather than a classical EMT process. In conclusion, our findings suggest that BAFF may play a critical role in promoting EMT and cell migration through JNK-Nrf2 signaling and highlighting its functional epithelial responses to viral mimetics, Poly (I:C) and the potential implications for respiratory diseases.
